# A four-microRNA panel in serum may serve as potential biomarker for renal cell carcinoma diagnosis

**DOI:** 10.3389/fonc.2022.1076303

**Published:** 2023-01-16

**Authors:** Rongkang Li, Wenkang Chen, Chong Lu, Xinji Li, Xuan Chen, Guocheng Huang, Zhenyu Wen, Hang Li, Lingzhi Tao, Yimin Hu, Zhengping Zhao, Zebo Chen, Liangchao Ni, Yongqing Lai

**Affiliations:** ^1^ Department of Urology, Guangdong and Shenzhen Key Laboratory of Reproductive Medicine and Genetics, Peking University Shenzhen Hospital, Clinical College of Anhui Medical University, Shenzhen, Guangdong, China; ^2^ The Fifth Clinical Medical College of Anhui Medical University, Hefei, Anhui, China; ^3^ Shantou University Medical College, Shantou, Guangdong, China

**Keywords:** microRNA, renal cell carcinoma, diagnosis, circulating biomarker, bioinformatics, logistic regression model

## Abstract

**Background:**

Renal cell carcinoma (RCC) is one out of the most universal malignant tumors globally, and its incidence is increasing annually. MicroRNA (miRNA) in serum could be considered as a non-invasive detecting biomarker for RCC diagnosis.

**Method:**

A total of 224 participants (112 RCC patients (RCCs) and 112 normal controls (NCs)) were enrolled in the three-phrase study. Reverse transcription quantitative PCR (RT-qPCR) was applied to reveal the miRNA expression levels in RCCs and NCs. Receiver operating characteristic (ROC) curves and the area under the ROC curve (AUC) were utilized to predict the diagnostic ability of serum miRNAs for RCC. Bioinformatic analysis and survival analysis were also included in our study.

**Results:**

Compared to NCs, the expression degree of miR-155-5p, miR-224-5p in serum was significantly upregulated in RCC patients, and miR-1-3p, miR-124-3p, miR-129-5p, and miR-200b-3p were downregulated. A four-miRNA panel was construed, and the AUC of the panel was 0.903 (95% CI: 0.847–0.944; p < 0.001; sensitivity = 75.61%, specificity = 93.67%). Results from GEPIA database indicated that CHL1, MPP5, and SORT1 could be seen as promising target genes of the four-miRNA panel. Survival analysis of candidate miRNAs manifested that miR-155-5p was associated with the survival rate of RCC significantly.

**Conclusions:**

The four-miRNA panel in serum has a great potential to be non-invasive biomarkers for RCC sift to check.

## Introduction

Renal cell carcinoma (RCC) is one of the malignant tumors from urinary tubular epithelium with high vascularization, which account for 80%–90% of renal malignancies (1). There were 431,288 people diagnosed, and 179,368 patients died with RCC worldwide in 2020 (2). Kidney cancer accounted for 76,080 new cases and about 13,780 people will die from it on 2021 in the USA (3, 4). What is worse, the morbidity of RCC is increasing steadily at an average rate of 3.7% per year (5). Although surge and biotherapy increase 5-year relative survival rate, the vascular invasiveness leads to early metastasis. Roughly 25% of patients with RCC present hematogenous metastasis to the bones, hepars, or lungs at the initial diagnosis (6–8). Therefore, prognosis of the curable RCC remains necessary for effective treatment.

In recent years, more and more small and early-stage RCCs were detected by the non-invasive radiological technologies like computed tomography (CT) and ultrasonography (US), which increased the positive proportion of potentially curable patients. However, there are still some limitations in the clinical application of imaging diagnosis, such as allergy to CT contrast agent, renal insufficiency, and the dependence of the accuracy of diagnosis on the experience of the doctor, which lead to unstable diagnosis (9, 10). In conclusion, one more stable method for assisting in the prediction of renal cell carcinoma is needed.

MicroRNAs (miRNAs) are evolutionarily short (~18–22 nucleotides), single-stranded, non-coding RNA molecules that could regulate gene through connecting the target mRNAs 3′-untranslated region (3′ UTR) (11). They regulate protein synthesis by inhibiting or promoting the transcription of messenger RNA. Cumulative studies indicated that miRNAs involve in the occurrence and inhibition of tumor, which means that the inhibition or overexpression of miRNAs could predict tumor occurrence (12, 13). Additionally, body fluids, such as serum or urine and plasma, could stably provide miRNAs. Consequently, miRNAs in serum have the latent capacity as a non-invasive tool for detecting tumor genesis (14, 15).

## Materials and methods

### Clinical specimens

In this study, a total of 224 participants from Peking University were randomly drawn, composed of 112 RCC patients and 112 normal controls from December 2017 to April 2021. All the patients were definitely diagnosed with RCC based on histopathological evaluation, who received no treatment. Correspondingly, inclusion criteria for the normal controls were men or women with no history of cancer and chronic illnesses. Peripheral blood, about 5–10 ml, from each participant was collected for serum extraction and processed it with centrifugation at 3,000 *g* for 10 min at 4°C in 2 h. Later, the processed serum was collected and stored in fresh tubes at −80°C. The present study was approved by the Ethics Committees of Peking University Shenzhen Hospital. In addition, participants’ characteristics are described in **Table 1**.

**Table 1:** Demographic and clinical manifestation of 224 participants (RCC and NCs).

	Training phase (n=60)		Validation phase (n=164)			
	RCC	NC	RCC		NC	
**Total number**	30	30		82	82	
**Age at diagnosis**			p=0.67			p=0.36
	51.8 ± 13.7	53.3 ± 12.7		50.0 ± 11.8	52.2 ± 18.6	
**Gender**			p=0.45			p=0.21
Male	14 (46.7%)	17(56.7%)		50 (61.0%)	42 (51.2%)	
Female	16 (53.3%)	13(43.3%)		32 (39.0%)	40 (48.8%)	
Location						
Left	18 (60.0%)			41 (50.0%)		
Right	12 (40.0%)			41 (50.0%)		
Fuhrman grade						
Grade I	4 (13.3%)			11 (13.4%)		
Grade II	15 (50.0%)			49 (59.8%)		
Grade III	10 (33.3%)			18 (22.0%)		
Grade IV	1 (3.3%)			4 (4.9%)		
AJCC clinical stage						
Stage I	20 (66.7%)			68 (82.9%)		
Stage II	7 (23.3%)			9 (11.0%)		
Stage III	2 (6.7%)			3 (3.7%)		
Stage IV	1 (3.3%)			2 (2.4%)		

Between the training phase and validation phase, there was no significant difference between RCC and NCs in age and gender. Parameters were shown as number (percentage). Statistical contrast was exerted through the Wilcoxon–Mann–Whitney test.

### Study design

The three-phase research was conducted to screen out candidate biomarkers and investigate and verify the effectiveness of miRNAs in detection and diagnosis. For Step 1, miRNAs related to RCC were selected as candidate miRNAs from researches published on the Gene Expression Omnibus and on the PubMed database. Then, the Encyclopedia of RNA Interactomes (ENCORI) database was applied for screening out the miRNA expression levels in the screening phase (16). These candidate miRNAs were chosen under the standard p-value of <0.01 and fold change (FC) of >2 or < –2 based on the expression level. In Step 2, 30 serum samples from RCCs and 30 from NCs were used to affirm the miRNAs with different expression level in the testing stage. For Step 3, another 82 RCC serum samples and 82 NC serum samples were utilized at the validation stage. To characterize the performance of miRNA to quantify the difference between RCCs and NCs, the receiver operating characteristic (ROC) curve analysis was performed to appraise the p-value and the area under the curve (AUC) to ascertain diagnostic ability of miRNAs. Finally, the ultimate model was designed through backward stepwise logistic regression method.

### RNA extraction, cDNA synthesis, and RT-qPCR

In order to control the variability in extraction and purification process, direct addition of 2 µl synthetic *Caenorhabditis elegans* miR-54 (cel-miR-54-5p) (10 nM/L, RiboBio, Guangzhou, China) into each serum sample done ahead of the procedure. Following the manufacturer’s guidance, total RNA from serum was extracted with TRIzol LS isolation kit (Thermo Fisher Scientific, Waltham, MA, USA) and lysed with 30 µl RNase-free water and stored at −80°C. After detecting its concentration with the help of a NanoDrop 2000 spectrophotometer (NanoDrop, Wilmington, DE, USA), we processed the amplification of miRNAs by using the specific primers of reverse transcription from Bulge-Loop miRNA qRT-PCR Primer Set. With Taqman probe on LightCycler 480 Real-Time PCR System (Roche Diagnostics, Mannheim, Germany), the real-time polymerase chain reaction was completed. The running program of RT-qPCR was set as follows: 95°C for 1 min, then 40 cycles of 95°C for 10 s, 55°C for 30 s, and 70°C for 30 s. The relative expression degree of target miRNAs were determined by using the 2^−△△Cq^ method (17).

### Bioinformatic analysis

The target genes corresponding to candidate miRNAs were screened out by MiRWalk3.0, and only the target genes associated with two or more candidate miRNAs could be chosen (18). Then, we used Enrichr database, a comprehensive gene that enabled gene set enrichment analysis to accomplish Gene Ontology (GO) functional annotation and Kyoto Encyclopedia of Genes and Genomes (KEGG) pathway enrichment analysis (19). To further explore the overall survival rate of RCC patients, we performed Kaplan–Meier survival analysis and the log-rank test of these candidate miRNAs with OncoLnc database (20).

### Statistical analysis

In our study, all statistical analyses were completed with the help of SPSS software (SPSS 20.0). Means and standard deviations (SDs) or number and percentage were calculated when comparing participants’ demographic and clinical characteristics using one-way univariate analysis of variance (ANOVA). For categorical variables, the Fisher’s exact test or the chi-squared was constructed to assess the statistical differences between groups. For continuous variables, the Kruskal–Wallis rank sum test or the one-way analysis of variance was constructed to assess the differences. The difference between the miRNA expression levels within RCCs and NCs was analyzed using Student’s t-test or the Mann–Whitney test. In addition, multiple logistic regression analysis was conducted for the establishment of miRNA signature. The predictive value of the miRNAs presented as ROC curves and the AUC for the diagnosis of RCC, and they were applied to appraise the diagnostic capability of serum miRNAs as follows: AUC of 0.5–0.7 (low), 0.7–0.85 (medium), and 0.85–1.0 (high). The optimal specificity and sensitivity were concluded by the Youden index (calculated as J = sensitivity + specificity − 1). p < 0.05 represented statistically significant.

## Result

### Study population

There were a total of 112 RCC patients and 112 normal controls involved in this study. No significant differences were observed among the experience group and the contrast group in the distribution of age and gender (p< 0.05). As shown in **Table 1**, it summed up the clinical pathological features of all participants.

### Screening out and testing candidate miRNAs

**Figure 1:**
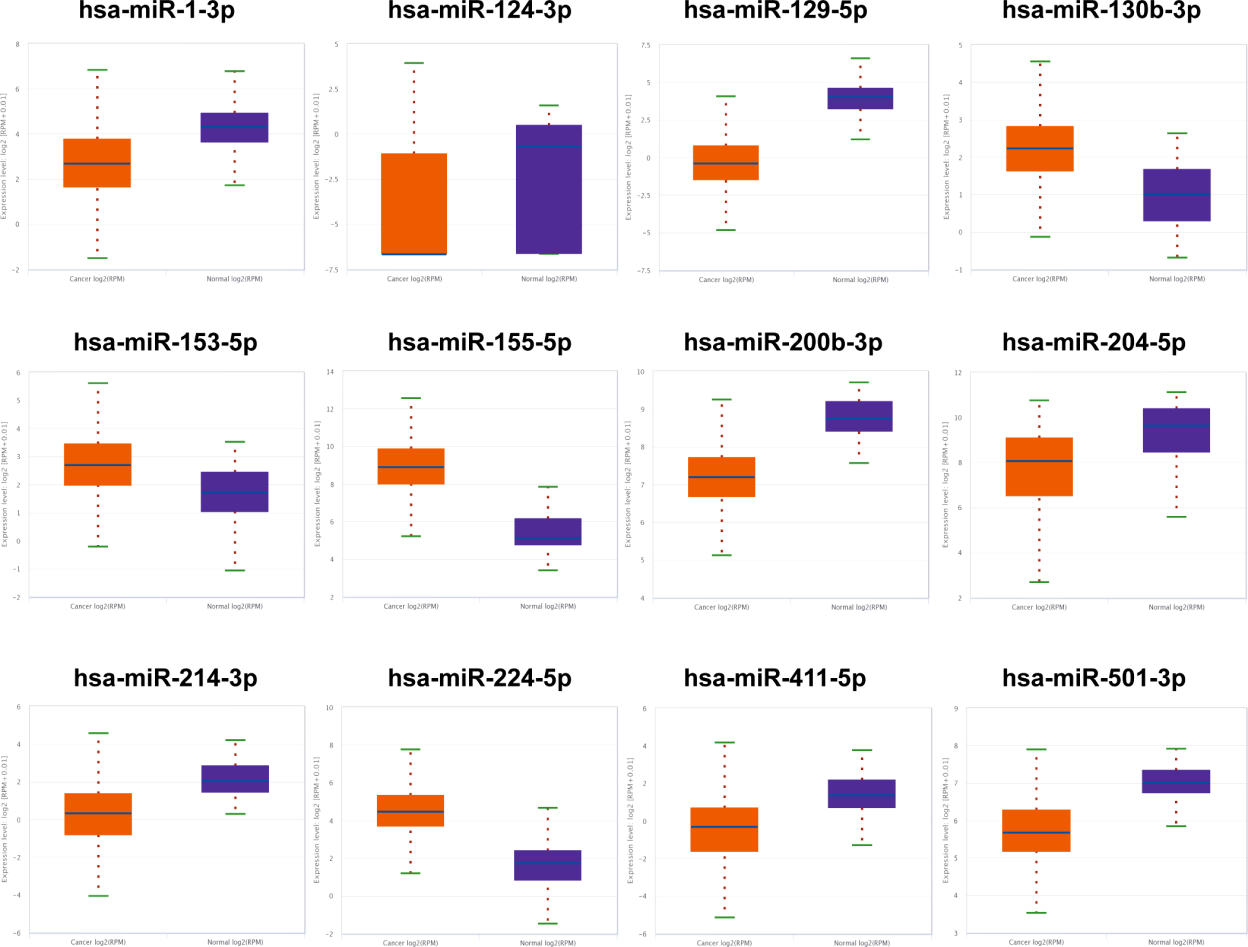
The 12 candidate miRNAs in ENCORI database. Based on the cutoff criterion: fold change (FC) >2 or <–2 and p-value <0.01, 12 miRNAs were screened out as the initial candidates.

The initial candidate miRNAs were sifted out through literature review in PubMed. Then, we accessed their expression levels in the ENCORI database and established the candidates’ miRNAs under the cutoff criteria p-value of < 0.01 and fold change (FC) of >2 or <–2 based on the expression level in 517 cancer and 71 normal tissues. Thus, 12 candidate miRNAs showing different expression levels between RCCs and NCs were split out (**Figure 1**); they would be tested in the next phase. Comparing the serum expression difference among RCCs and NCs, four miRNAs (hsa-miR-130b-3p, hsa-miR-153-5p, has-miR-155-5p, and has-miR-224-5p) were overexpressed and the other miRNAs (has-miR-1-3p, has-miR-124-3p, has-miR-129-5p, has-miR-200b-3p, hsa-miR-204-5p, hsa-miR-214-3p, hsa-miR-411-5p, and has-miR-501-3p) were downregulated in RCC patients.

**Figure 2:**
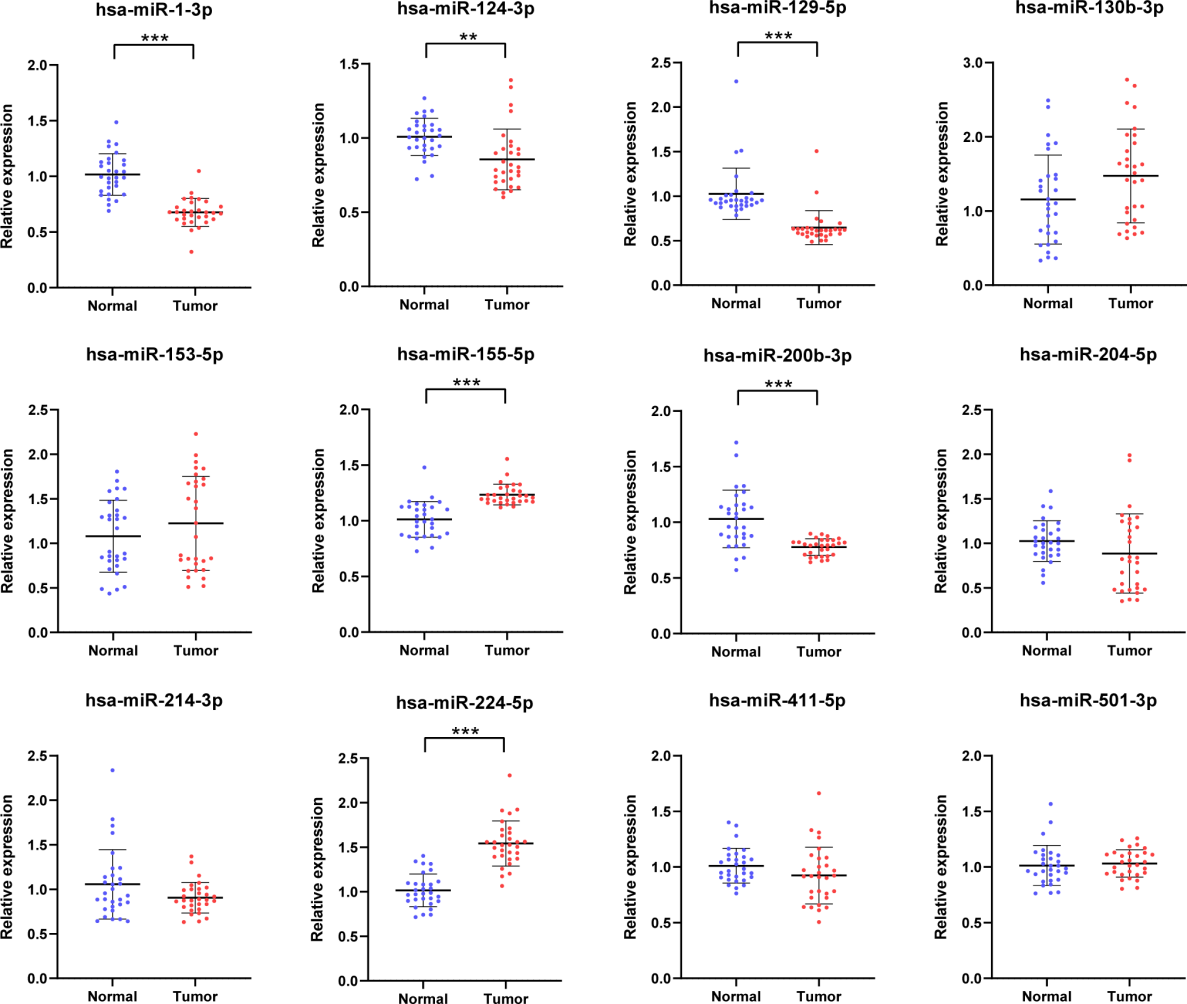
Relative serum expression levels of 12 candidate miRNAs. There were a total of 30 RCCs serum samples and 30 NC serum samples utilized in this phrase and 6 miRNAs ultimately showed significant difference. **p < 0.01, ***p < 0.001.

The 12 candidate miRNAs were further confirmed with 30 NCs and 30 RCC patients by means of qRT-PCR analysis. As shown in **Figure 2**, six miRNAs (miR-1-3p, miR-124-3p, miR-129-5p, miR-155-5p, miR-200b-3p, and miR-224-5p) ultimately showed statistically significant difference in serum expression degree.

### Validation and diagnostic capability of the candidate miRNAs

**Figure 3:**
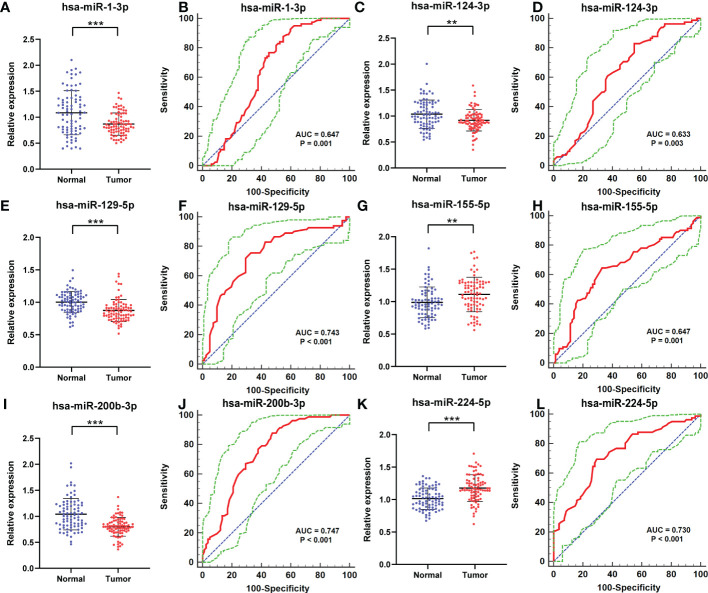
Relative expression counting and receiver operating characteristic curve (ROC) analyses in the validation phase of six elected miRNAs. This phase consisted of 82 RCCs and 82 NC serum samples. In RCC, the miRNAs with higher expression were miR-155-5p **(G)** and miR-224-5p **(K)**; their AUCs are 0.647 (95% CI: 0.568–0.720; p= 0.001; **(H)** and 0.730 (95% CI: 0.655–0.796; p < 0.001; **(L)**. Significantly lower expressed miRNAs were as follows: miR-1-3p **(A)**, miR-124-3p **(C)**, miR-129-5p **(E)**, and miR-200b-3p **(I)**. Their AUCs are 0.647 (95% CI: 0.569–0.720; p =0.0001; **(B)**, 0.633 (95% CI: 0.554–0.706; p = 0.003; **(D)**, 0.743 (95% CI: 0.669–0.808; p< 0.001; **(F)**, and 0.747 (95% CI: 0.674–0.812; p< 0.001; **(J)**. **p < 0.01, ***p < 0.001.

To further identify whether or not the plasma levels of miR-1-3p, miR-124-3p, miR-129-5p, miR-155-5p, miR-200b-3p, and miR-224-5p show difference in RCC patients, qRT-PCR was conducted to affirm the expression of the selected miRNAs in participant serum. The six candidate miRNAs elected from the testing phase were analyzed on another 82 RCCs and 82 NCs at the validation phase. As a result, all of them finally show relative dysregulated with p-value <0.05 (**Figure 3**). The result show that expression of miR-155-5p and miR-224-5p is upregulated compared with NCs, and the other is opposite.

Furthermore, the ROC curves were performed to figure out the diagnostic capability as biomarkers in RCC prognosis of these six candidate miRNAs. The respective areas under the curves for miR-1-3p, miR-124-3p, miR-129-5p, miR-155-5p, miR-200b-3p, and miR-224-5p were 0.647 (95% confidence interval (CI): 0.569–0.720; p =0.0001; **Figure 3B**),0.633 (95% CI: 0.554–0.706; p = 0.003; **Figure 3D**), 0.743 (95% CI: 0.669–0.808; p< 0.001; **Figure 3F**), 0.647 (95% CI: 0.568–0.720; p= 0.001; **Figure 3H**), 0.747 (95% CI: 0.674–0.812; p< 0.001; **Figure 3J**), and 0.730 (95% CI: 0.655–0.796;p < 0.001; **Figure 3L**). Moreover, Youden index was performed to calculated optimum cutoff values, and specific sensitivity and specificity of these six candidate miRNAs in RCC diagnosis are shown in **Table 2**.

**Table 2:** Outcomes of receiver operating characteristic curves and Youden index for six candidate miRNAs and the panels.

	AUC	p-value	95% CI	Associated criterion	Sensitivity (%)	Specificity (%)
miR-1-3p	0.647	0.0010	0.569–0.720	≤1.15	87.80	45.12
miR-124-3p	0.633	0.0026	0.554–0.706	≤1.04	82.93	45.12
miR-129-5p	0.743	<0.001	0.669–0.808	≤0.92	71.95	70.73
miR-155-5p	0.647	0.0009	0.568–0.720	>1.03	64.63	67.09
miR-200b-3p	0.747	<0.001	0.674–0.812	≤0.98	87.80	52.44
miR-224-5p	0.730	<0.001	0.655–0.796	>1.1	69.51	70.73
four-miRNA panel	0.903	<0.001	0.847–0.944	>0.62787	75.61	93.67

AUC, area under curve; CI, confidence interval.

### Establish a composite miRNA panel for diagnosing RCC better

**Figure 4:**
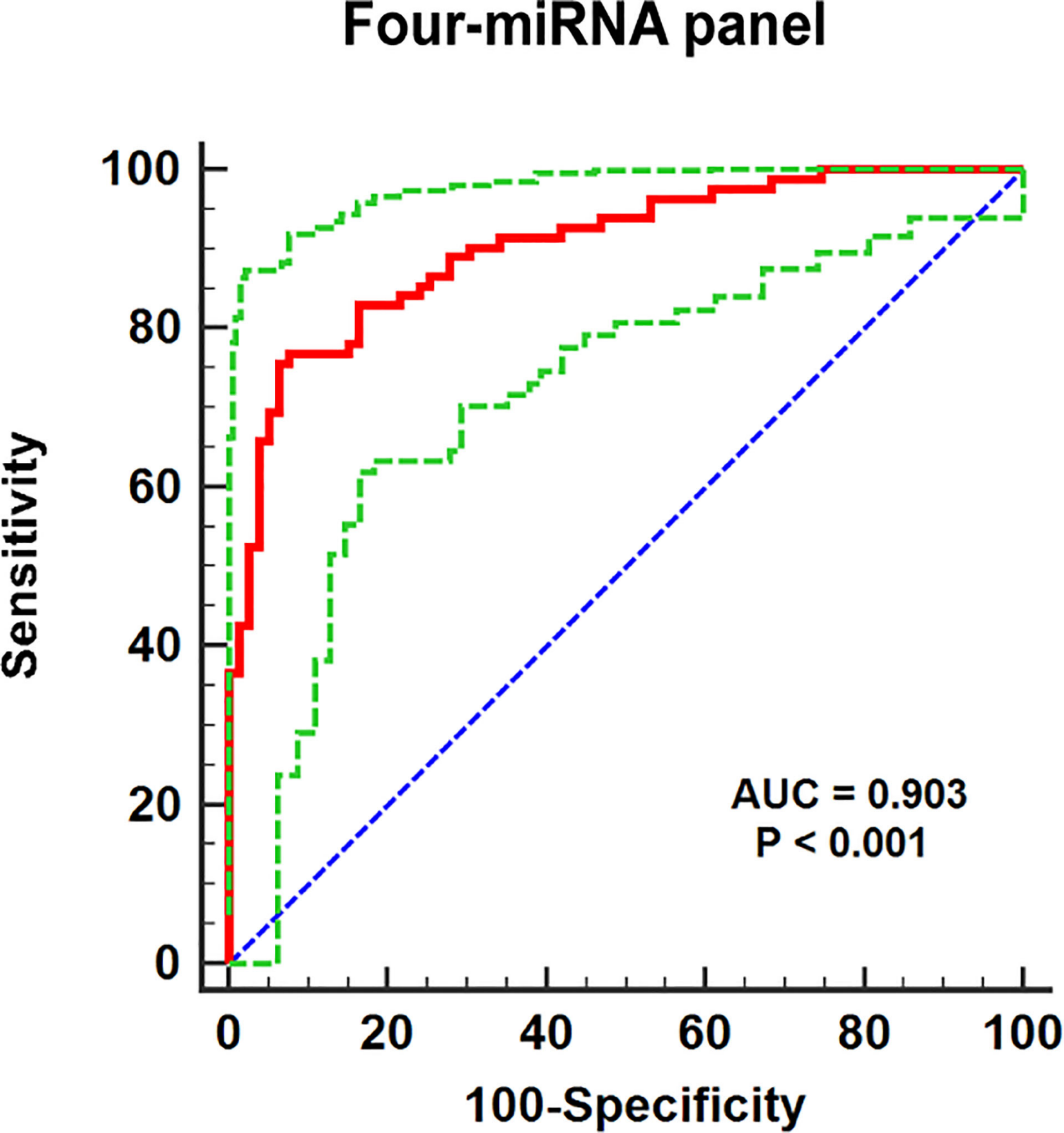
The ROC curve evaluation for the four-miRNA panel. This four-miRNA panel contained miR-1-3p, miR-155-5p, miR-200b-3p, and miR-224-5p, and the AUC for the panel was 0.903 (95% CI: 0.847–0.944; p< 0.001; sensitivity = 75.61%, specificity = 93.67%).

In addition, a logistic regression model was established for further enhancement of early diagnostic ability in RCC, while combining several miRNAs may contribute to better diagnostic ability than separate miRNA. It indicated that making up miR-1-3p, miR-155-5p, miR-200b-3p, and miR-224-5p, as the combined biomarker emerged the best panel to screening RCC. As shown in **Figure 4**, the AUC for four-miRNA panel is 0.903 (95% CI: 0.847–0.944; p< 0.001; sensitivity = 75.61%, specificity = 93.67%). The formula constructed the miRNA panel was as follows: Logit(p) = −1.997 −3.181 × miR-1-3p − 5.889 × miR-200b-3p + 2.643 × miR-155-5p + 7.100 × miR-224-5p.

### Bioinformatic analysis of candidate miRNAs

**Figure 5:**
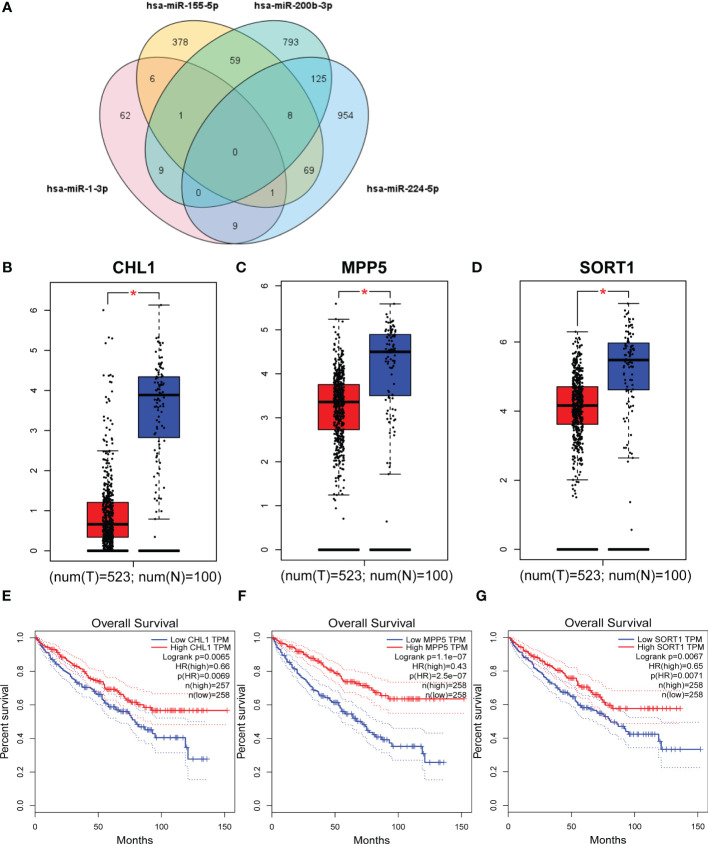
Target genes and overall survival of target genes. Genes that were predicted in over two miRNAs were regarded as potential targets, and eventually, 278 genes were elected **(A)**. GEPIA was applied to predict these genes relating to four candidate miRNAs in 523 RCCs and 100 NCs. CHL1 **(B)**, MPP5 **(C)**, and SORT1 **(D)** were dysregulated with |log2FC| > 1and p < 0.01. CHL1 **(E)**, MPP5 **(F)**, and SORT1 **(G)** were associated with the prognosis of RCC. T, tumor; N, normal control. *p <0.01.

We predicted target gene candidates for each miRNAs using miRWalk3.0, and a total of 278 genes were elected as target genes with the criteria of which gene predicted in more than two miRNAs (**Figure 5A**). Then, under the criteria, |log2FC| > 1, p < 0.01, results from GEPIA database indicated that CHL1, MPP5, and SORT1 could be regarded as the latent target genes for four-miRNA panel (**Figures 5B–D**), whose expression levels are significantly different between RCCs and NCs. In addition, data from GEPIA database demonstrated that obvious association was found between CHL1, MPP5, and SORT1 expression and the prognosis of RCCs in overall survival (**Figures 5E–G**).

**Figure 6:**
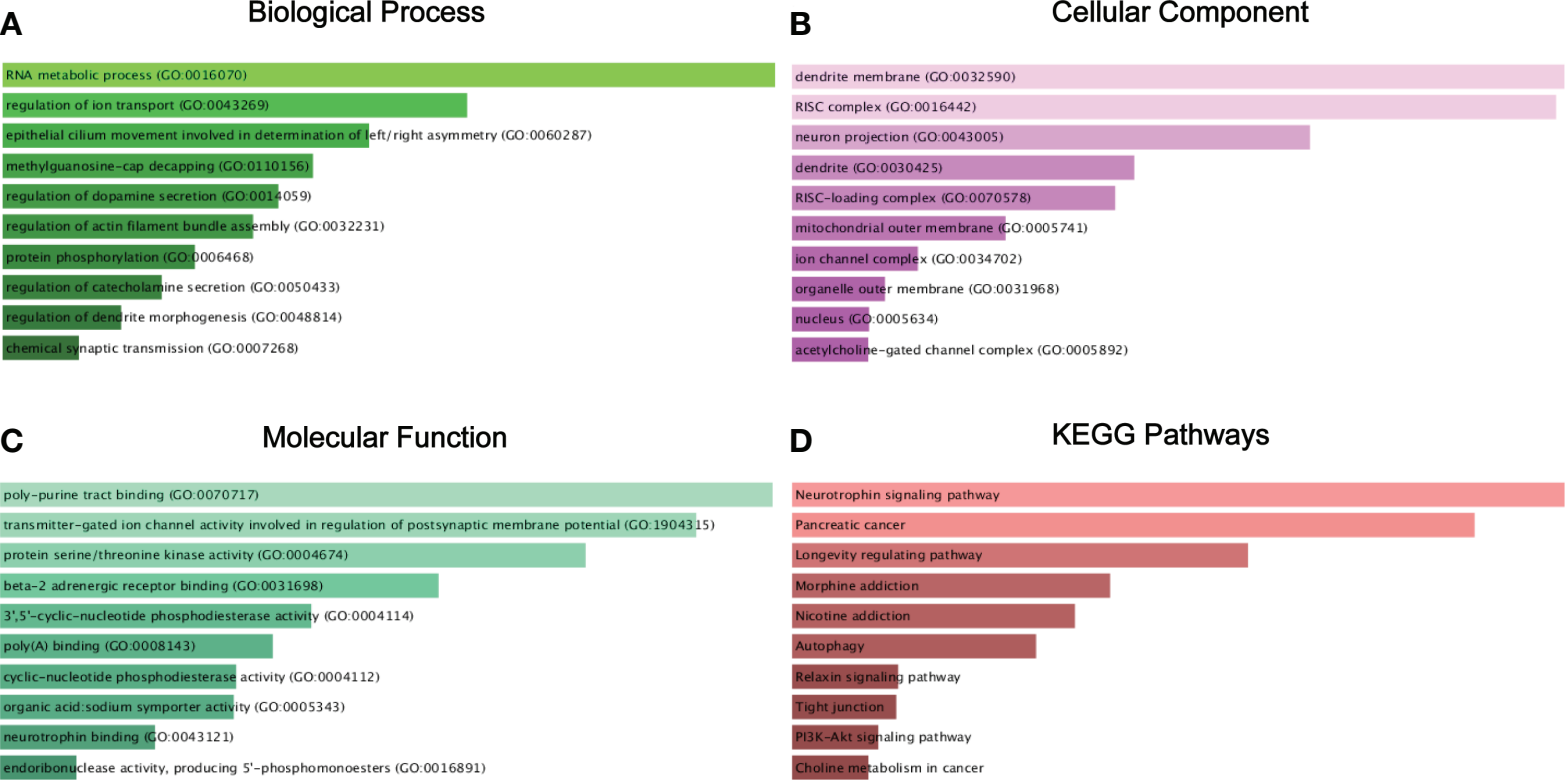
Target genes GO functional annotation and KEGG pathway enrichment analysis. Biological process (BP) analysis **(A)**, cellular component (CC) analysis **(B)**, molecular function (MF) analysis **(C)**, and KEGG pathway enrichment analysis **(D)**.

For GO annotation and KEGG pathway enrichment analysis, we put the 278 targeted genes into the Enrichr database. As shown in **Figures 6A–C**, the top 3 most counted enriched GO functional annotation categories for the biological process category, cellular component category, and molecular function category were GO:0016070 (RNA metabolic process), GO:0043269 (regulation of ion transport), GO:0060287 (epithelial cilium movement involved in determination of left/right asymmetry) and GO:0070717 (poly-purine tract binding), GO:1904315 (transmitter-gated ion channel activity involved in regulation of postsynaptic membrane potential), GO:0004674 (protein serine/threonine kinase activity) and GO:0032590 (dendrite membrane), GO:0016442 (RISC complex), and GO:0043005 (neuron projection), respectively. KEGG pathway analysis demonstrated various pathways that the target genes enriched in, including neurotrophin signaling pathway, pancreatic cancer, and longevity regulating pathway (**Figure 6D**).

### Survival analysis of candidate miRNAs

**Figure 7:**
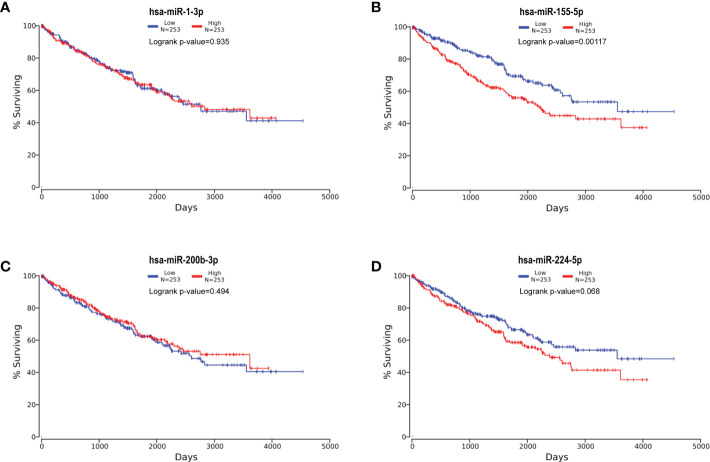
Kaplan–Meier survival curves of four candidate miRNAs. Kaplan–Meier survival curves of hsa-miR-1-3p **(A)**, hsa-miR-155-5p **(B)**, hsa-miR-200b-3p **(C)**, and hsa-miR-224-5p **(D)**. The analysis manifested that miR-155-5p significantly associated with RCC survival rate, and RCCs with higher miR-155-5p expression level tended to have worse prognosis.

Subsequently, based on dichotomized QPCT expression by a log-rank test, we compared RCCs survival with the help of Kaplan–Meier survival analysis and data from OncoLnc database with 506 RCC patients. The analysis manifested that a significant association existed among miR-155-5p and the survival rate of RCC, and RCCs with higher miR-155-5p expression level tended to have worse prognosis (**Figure 7**).

## Discussion

Renal cell carcinoma originates from renal tubular epithelial cells and is a lethal urological malignancy, representing a comprehensive 90% of all renal carcinoma, with approximately 295,000 new cases diagnosed annually and about 134,000 cases of cancer-related death per year. As advanced RCC is a fatal disease with only 11.7% 5-year survival rate (21), the effective prediction methods matter. Presently, the main diagnostic means are imaging examination, renal puncture biopsy, etc. However, CT and MRI could reach a higher predictive accuracy only in the high-grade RCC, which means that imaging examination’s early-grade RCC prediction ability is limited (22–24). Invasive and high cost leads to less universality for RCC patients. Therefore, one more stable prognosis methods of RCC is needed, such as miRNA.

In the present study, an investigation of serum miRNA expression was conducted in RCC patients and normal controls using RT-qPCR. Six miRNAs were related to the RCC, namely, miR-1-3p, miR-124-3p, miR-129-5p, miR-155-5p, miR-200b-3p, and miR-224-5p, and the analysis also show that expression of miR-155-5p and miR-224-5p was upregulated and miR-1-3p, miR-124-3p, miR-129-5p, and miR-200b-3p were downregulated, respectively. For further verification of the diagnostic ability in RCC, we made up miR-1-3p, miR-155-5p, miR-200b-3p, and miR-224-5p as a combined biomarker, which emerged as the best panel to screen RCC. As a result, our research revealed that miR-200b-3p was the strongest effective independent predictor among six candidates mentioned before.

Compared with NCs, miR-200b-3p was downregulated in RCC serum, which means that it could show repression in tumor development. In ovarian cancer and hepatocellular carcinoma, for instance, some studies demonstrated that the miR-200 family is a significant player in invasion and migration in a variety of cancer and vascular complications (25, 26). Pecot et al. claimed that miR-200b-3p may act as antioncogene through angiogenesis blocking (27). In addition, some studies revealed that miR-200b-3p could participate in improving treatment benefit, especially increases in response to microtubule-targeting agents (25, 28). The overexpression of miR-200b-3p may be associated with low expression of β-tubulin III and improve the effectiveness of paclitaxel chemotherapy. Furthermore, Chang et al. indicated that miR-200 absence leads to docetaxel resistance in RCC (29). Thus, miR-200b-3p could inhibit tumor development and improve chemosensitivity.

The overall survival of RCC patient was compared by Kaplan–Meier survival analysis, and their analysis manifested that miR-155-5p had significant connection with RCC survival rate. In addition, miR-155-5p was observed to play a role as proinflammatory factor, which can increase IL-1β, IL-6, IL-8, and tumor necrosis factor (TNF) production, and antitumor immunity could increase the miR-155-5p serum expression level (30, 31). In addition, in ovarian cancer cells, Xiang Li et al. indicated that exo-miR-155-5p release could be promoted by ROS inhibition, resulting in the elevation of miR-155-5p, which means that miR-155-5p downregulated expression could create the tumor growth favoring microenvironment (32). Therefore, RCC patients with higher miR-155-5p expression level tended to have worse prognosis.

For RCC screening, miR-224-5p was illustrated to act as a biomarker in our study. Previous research demonstrated that miR-224-5p and miR-1-3p played a role in the process of RCC. The miR-224-5p expression degree in urinary extracellular vesicles (EVs) was also overexpressed in RCCs, and miR-224-5p in EVs regulated PD-L1 *via* inhibiting cyclin D1 in RCC progression (33). Through miR-224-5p/CHSY1 axis, LINC01094 activated by FOXM1 played its tumor-promoting role in the development of CCRCC (34). Same as in serum, the miR-1-3p expression levels in RCC cell lines and tissues were significantly suppressed, and miR-1-3p reduced fibronectin 1 to restrain the epithelial–mesenchymal transition process in RCC (35, 36).

Our study result showed that CHL1, MPP5, and SORT1 could be considered as potential target genes. CHL1 is an inhibitory oncogene in many tumors, which is involved in the inhibition of cancer cell proliferation, epithelial–mesenchymal transition (EMT), and even chemotherapy resistance, like esophageal squamous cell carcinoma and renal carcinoma (37–40). Furthermore, a previous study claimed that CHL1 acted as an independent and unfavorable prognostic factor for the overall survival rate of CCRCCs and suggested that lower expression of CHL1 leads to poor overall survival rate (41). Furthermore, knocking out CHL1 could promote the senescence and apoptosis of glioma by combining receptor composed of Bax, Bcl-2, and caspase-3 (42). Yang et al. indicated that in patients with depression, CHL1 in CD4+ T cells and CD8+ T cells increased and its expression level decreased significantly after treatment, which may prove that CHL1 affects the pathogenesis and treatment of depression through CD4+ T cells and CD8+ T cells (43). Other biomedical analysis suggested that CHL1 may be related to CD8+ T cells and macrophage M2 cells in patients with ccRCC, but it lacked biomolecule experiment, which could be the inspiration for further experiment (44). It is reported that SORT1 gene is a member of vacuolar protein sorting 10 protein-related family, and it can encode Sortilin protein. With further research on SORT1, it could be a promising tumor target, and more and more cancer tissues show overexpression of SORT1 (45–47). SORT1 functions could conjugate cytotoxic agents, like Docetaxel/Doxorubucin, and accurately and specifically inhibit growth of tumor (48). Miyakawa et al. indicated that anti-SORT1 could inhibit SORT1 to increase the level of PGRN in the cerebrospinal fluid, which is beneficial to the prognosis of frontotemporal dementia (FTD) (49). However, the function of PGRN has not been fully clarified, so we can verify whether it participates in the immune process of RCC occurrence or apoptosis in the next experiment (50, 51). MPP5 belongs to the membrane-associated guanylate kinase family and is responsible for establishing mammalian cell polarity, which is essential in tissue organization. Cell polarity plays a fundamental role in the epithelial tissue architecture and function, and it could regulate the cell growth and division. Therefore, loss of cell polarity triggers cancer progression and organ dysfunction, which means that the loss of MPP5 is a hallmark of cancer (52–54). MPP5 is crucial for the nervous system developing and bladder cancer progressing (55, 56). However, the present studies on MPP5 are still rare, and the mechanism affecting tumor progression is not clear yet.

## Conclusions

In conclusion, we identified several differentially expressed serum miRNAs (miR-1-3p, miR-124-3p, miR-129-5p, miR-155-5p, miR-200b-3p, and miR-224-5p) among RCCs and NCs. CHL1, MPP5, and SORT1 could be considered as potential target genes. However, the mechanism of their role in different tumors is still unclear. There are few studies on some genes in RCC that lead to rare understanding of different functional manifestations in different tumors. If further research can be carried out, they could play a greater role in predicting tumorigenesis.

## Data availability statement

Publicly available datasets were analyzed in this study. This data can be found here: https://starbase.sysu.edu.cn/index.php and https://www.ncbi.nlm.nih.gov/geo/.

## Ethics statement

The studies involving human participants were reviewed and approved by Ethics Committee of Peking University Shenzhen Hospital. The patients/participants provided their written informed consent to participate in this study. Written informed consent was obtained from the individual(s) for the publication of any potentially identifiable images or data included in this article.

## Author contributions

Conceptualization: LN and YL. Data curation: CL, XL, and XC. Formal analysis: RL and WC Investigation: RL and WC. Methodology: RL and WC. Project administration: RL and WC. Validation: GH, ZW, and HL. Visualization: LT, YH, ZZ, and ZC. Writing—original draft: RL and WC. Writing—review and editing: all authors. All authors contributed to the article and approved the submitted version.
